# Degradation rate uniformity determines success of oscillations in repressive feedback regulatory networks

**DOI:** 10.1098/rsif.2018.0157

**Published:** 2018-05-09

**Authors:** Karen M. Page, Ruben Perez-Carrasco

**Affiliations:** Department of Mathematics, University College London, Gower Street, WC1E 6BT London, UK

**Keywords:** oscillations, repressilator, degradation rates, dynamical systems, gene regulatory networks

## Abstract

Ring oscillators are biochemical circuits consisting of a ring of interactions capable of sustained oscillations. The nonlinear interactions between genes hinder the analytical insight into their function, usually requiring computational exploration. Here, we show that, despite the apparent complexity, the stability of the unique steady state in an incoherent feedback ring depends only on the degradation rates and a single parameter summarizing the feedback of the circuit. Concretely, we show that the range of regulatory parameters that yield oscillatory behaviour is maximized when the degradation rates are equal. Strikingly, this result holds independently of the regulatory functions used or number of genes. We also derive properties of the oscillations as a function of the degradation rates and number of nodes forming the ring. Finally, we explore the role of mRNA dynamics by applying the generic results to the specific case with two naturally different degradation timescales.

## 1. Introduction

Genetic regulatory networks (GRNs), consisting of the interactions between a set of genes, are core to the regulation of the temporal genetic expression profiles required for various cellular processes, ranging from cell fate determination during embryogenesis to cellular homesotasis [1–6]. GRNs are capable of many dynamical functions, including oscillatory gene expression [7], as has been observed in somitogenesis [8], circadian clocks [9], the activity of the p53 tumour suppressor [10,11] or the nuclear factor *κ*B localization [12].

Owing to their range of utilities, different oscillatory gene regulatory circuits have been synthetically engineered [13]. In particular, a lot of attention has been focused on the engineering of ring oscillators consisting of a set of genes interacting with each other sequentially and forming a repressive feedback loop. This work was initiated by the synthesis of the three-gene repressilator [14], that has been further refined to improve its oscillation properties (e.g. [15,16]). Consequently, the theoretical and numerical analysis of the working of ring oscillators has also received substantial attention. Such work was pioneered by Fraser & Tiwari [17] who performed numerical simulations. Subsequent analysis showed that for sufficiently strong repression, oscillations arise due to a Hopf bifurcation, relating the genetic oscillatory behaviour with dynamical systems theory [18], which has led to many different studies delving into the dynamical properties of the oscillations (e.g. [19–23])

These analytical and numerical studies of biochemical circuits require insight into a set of simultaneous nonlinear feedback interactions between multiple genes usually analysed as a set of ordinary differential equations (ODEs). Determining the role of different parameters in the solutions to these equations poses enormous analytical complexity that hinders quantitative studies. For this reason, computational and analytical studies are often reduced to tackling relatively small networks, and, even in such cases, to a reduced parameter set or certain simplified regulatory functions. This can constrain the range of applications of the results found [24]. Even in the case of the repressilator, the dynamical complexity can be huge [25] and restrictive assumptions within the quantitative model again become unavoidable. This highlights the necessity to develop tools capable of understanding the dynamical properties of the system independently of the regulatory functions used.

A useful assumption, present in the vast majority of studies, is that the degradation rates of proteins are identical for different genes. However, due to the high span of protein structures and mechanisms controlling degradation rates, such as ubiquitination [26,27], the turnover rate can range orders of magnitude in the proteome of a single system [28,29]. As oscillations in a network are generated by an ongoing imbalance between the production and degradation of the different species, it is expected that degradation rates play a determinant role in the behaviour of oscillatory circuits. Particularly, simulations of a repressilator model showed that oscillations are favoured for comparable values of the degradation of the protein and mRNA [14], and, more generally, a certain level of symmetry around the ring [30]. Nevertheless, there is no analytical study that gives insight into the role of degradation rates for general ring oscillators independent of the regulatory functions used.

To gain insight into the role of degradation rates in oscillatory networks, dynamical systems theory and bifurcation theory have proved to be essential tools. These allow us to categorize different possible dynamical responses of oscillatory networks [7,18,31,32]. Using bifurcation theory, we aim to obtain information on the role of degradation rates in oscillatory networks, making these results as general as possible and using minimal details of the regulatory functions. Specifically, we show how relevant information on the interactions between different genes can be captured with a single parameter. We show how this parameter controls the appearance of oscillations through a Hopf bifurcation. First, we develop our methodology for the repressilator, expanding the theory in the following sections to negative feedback ring oscillators with an arbitrary number of species. Finally, we study the case in which the species are categorized as mRNAs and proteins, which have distinct degradation rates, giving insight into the role of mRNA dynamics in the performance of ring oscillators.

## 2. Results

### 2.1. Three-gene repressilator

**Figure 1.**
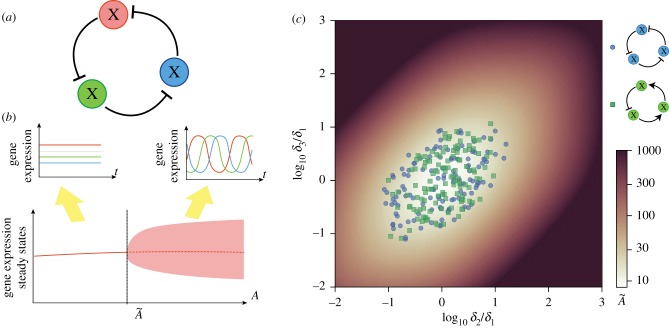
Oscillatory behaviour of the repressilator. (*a*) Schematic of the repressilator. (*b*) Bifurcation diagram schematic shows how the oscillations appear and disappear through a Hopf bifurcation depending on the magnitude *A* that summarizes the negative feedback strength of the circuit. (*c*) Degradation rate relationship of repressilator networks showing oscillations from a random screening (squares and circles). Different symbols stand for the screening of the repressilator (blue circles), and the repression ring with only one repression and two activations (green squares). Random repressilator networks were generated by sampling random parametrizations of *f*_*i*_ and sampling relative degradation rates covering the whole square plotted (*δ*_2_/*δ*_1_ and *δ*_3_/*δ*_1_ between [10^−2^, 10^3^], *δ*_1_ = 1 in all simulations); as expected by the analysis, all the successful oscillators concentrate in the zone with similar degradation rates. Results are compared with the dependence of *Ã* with degradation rates from equation (2.5) (brown shading). Random interaction functions were generated using the thermodynamic function *f*_*i*_(*x*) = *a*_*i*_(1 + *ρ*^*R*^_*i*_(1 + *x*/*k*_*i*_)^*h*^)^−1^ for the repressions and *f*_*i*_(*x*) = *a*_*i*_(1 + *ρ*^*A*^_*i*_[(1 + *x*/*k*_*i*_)/(1 + *l*_*i*_*x*/*k*_*i*_)]^*h*^)^−1^ for the activations with *h* = 3. Random parameters were sampled logarithmically from the intervals *k*_*i*_ : [10^−9^, 10^−5^], *a*_*i*_ : [10^−4^, 10^4^], *ρ*^*R*^_*i*_ : [10^−4^, 10^4^], *ρ*^*A*^_*i*_ : [10^3^, 10^11^], *l*_*i*_ : [10, 10^5^].

The classic general form of the repressilator consists of three genes repressing each other sequentially [14] (figure 1*a*). In the simple case in which mRNA dynamics are considered fast compared with protein dynamics, the dynamical evolution of the system can be described as a set of ODEs
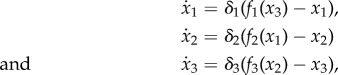
where *f*_1_, *f*_2_ and *f*_3_ describe the repressive interactions between genes and are therefore decreasing, positive functions. *f*_*i*_ can be thought of as the maximal expression level of gene *i* multiplied by the probability that its repressor is inactive. At any given steady state (x^*^_1_, x^*^_2_, x^*^_3_) given by 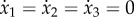, the repressilator follows the relationship x^*^_3_ = *f*_3_(*f*_2_(*f*_1_(x^*^_3_))) ≡ *F*(x^*^_3_), where the function *F* captures the overall negative feedback. As *F*(*x*) is a decreasing, positive function, there is a unique possible value of x^*^_3_, which yields unique values x^*^_1_ = *f*_1_(x^*^_3_) and x^*^_2_ = *f*_2_(x^*^_1_). The stability of the protein levels dictated by this steady state, can be computed through the eigenvalues of its Jacobian matrix2.1
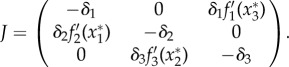

These eigenvalues λ satisfy the characteristic equation2.2
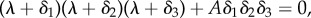
where *A*≡−*f*′_1_(x*_3_)*f*′_2_(x*_1_)*f* ′_3_(x*_2_) =−*F*′(x*_3_) is the modulus of the slope of the composite repression function at the steady state. Interestingly, the parameter *A* contains all the details of the interactions of the network necessary to solve the characteristic equation (2.2). This means that the eigenvalues of the characteristic equation and so the stability of the steady protein levels will depend only on *A* and on the degradation rates. This allows us to perform the stability analysis without any further information on the explicit form of the repressive interactions. Concretely, since *F*(*x*) is a monotonically decreasing function (*A* > 0) the product of the eigenvalues of *J* will always be negative,2.3
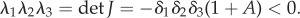
Therefore, the repressive ring forbids any eigenvalue to be zero. As a result, a change in stability of the steady state can only occur through a Hopf bifurcation, in which a pair of complex conjugate eigenvalues crosses the imaginary axis. We write this pair 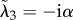 and 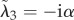, where *α* is the angular velocity of the sustained oscillations that appear at the Hopf bifurcation. Following equation (2.3), the other eigenvalue λ_1_ must be real and negative, everywhere, and in particular at the Hopf bifurcation (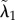).

Introducing the purely imaginary eigenvalues 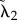 and 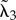 in the characteristic equation (2.2), expressions for *α* and 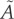 (value of *A* at the bifurcation) are obtained that only depend on the degradation rates,2.4
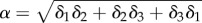
and2.5
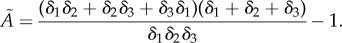
As the value of 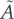 is unique, the repressilator has a single Hopf bifurcation with gene expression (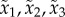). Concretely, at the lowest value of *A* (*A* = 0), the eigenvalues of the Jacobian are all negative (λ_*i*_ = −*δ*_*i*_,  *i* = {1, 2, 3}), and the steady state is stable. As there is a change in the stability of the steady state at 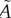, the steady state is stable for *A* < 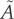 and unstable (with the appearance of a stable oscillatory orbit) for *A* > 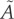. Thus, the smaller the value of 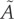, the easier it is to find oscillations in the system (figure 1*b*). Strikingly, the value of 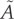 just depends on the degradation rates (see equation (2.5)) and is minimized when they are equal, *δ*_1_ = *δ*_2_ = *δ*_3_, giving 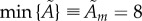. Therefore, the closer the degradation rates are to being equal, the less strict is the condition on the network parameters through *A* in order for the system to oscillate. This has been tested computationally by generating random repressilator networks, showing that knowledge of the value of 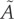
_*m*_ derived from the degradation rates gives a prediction of the propensity for oscillations of the repressilator network (figure 1*c*).

It is interesting to note that the three-gene repressilator analysis extends straightforwardly to the negative feedback loop case consisting of two activations and one repression. In this case, the composite function *F* is again a positive decreasing function which is the only requirement in our analysis, yielding exactly the same results (figure 1*c*).

### 2.2. *N*-component negative feedback ring

The reduced three-gene scenario considered above does not include intermediate mRNA dynamics or other intermediate regulatory steps. Additionally, it is not straightforward to apply the results to repressive rings with a gene number greater than three, such as the artificial circuits created by Niederholtmeyer *et al*. [16]. To analyse these systems, we can extend the repressilator by considering the general case of *N* biochemical species as an *N*-dimensional monotone cyclic feedback system [33]:2.6
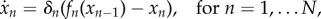
where *x*_0_ ≡ *x*_*N*_. To ensure that the network presents a negative feedback loop, it must contain an odd number of repressions *N*_*R*_, i.e. *N*_*R*_ of the functions *f*_*n*_ are monotonic decreasing positive functions. In addition there are *N*_*I*_ = *N* − *N*_*R*_ activations, i.e. *N*_*I*_ of the functions *f*_*n*_ are monotonic increasing positive functions. To extend the result to *N* components, we will follow a derivation equivalent to that of the repressilator. In this case, the steady state is located at x*_N_ = *f*_*N*_(*f*_*N*−1_(….*f*_1_(x*_N_))) ≡ *F*(x*_N_). As in the three-gene repressilator, *F*(*x*) is a positive monotonically decreasing function and so there is a single value for x*_N_ and hence a unique steady state x*_1_ = *f*_1_(x*_N_), x*_2_ = *f*_2_(x*_1_), …, x*_N−1_ = *f*_*N*−1_(x*_N−2_).

As the *N*-component repressive ring cannot show chaotic behaviour (see the electronic supplementary material), when the steady state is unstable, it will not be able to attract trajectories, and orbits will converge to a limit cycle where all the biochemical species will oscillate in time. As in the repressilator, this allows us to study the oscillatory properties of the GRN through its Jacobian *J* at the steady state x*2.7
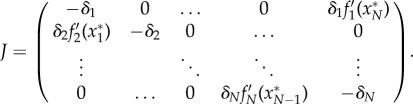
The corresponding characteristic equation is given by
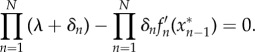
As in the three-gene case, the chain rule for differentiation gives us 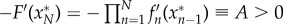 and hence the characteristic equation can be written as2.8
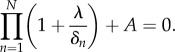
Therefore, as with the classic repressilator, despite all the potential complexity in the repression functions of the network, the stability of the unique steady state only depends on the degradation rates and the parameter *A*, which gathers information on the global negative feedback loop, as the modulus of the slope of the composite repression function of a gene on itself at the steady state. The product of the eigenvalues of *J* also follows the same pattern as equation (2.3),2.9
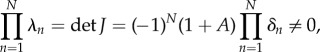
forbidding a zero eigenvalue of *J*, so the steady state can only lose stability via a Hopf bifurcation (see the electronic supplementary material). At *A* = 0, the Jacobian matrix has eigenvalues λ =−*δ*_*n*_ for *n* = 1, …, *N*, and is therefore a stable node. Increasing *A* away from zero, oscillations arising through a Hopf bifurcation will appear at the smallest value *A* = 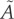 at which *J* has a pair of imaginary eigenvalues. Letting the pair of eigenvalues be ±*iα* with the angular velocity *α* > 0 and introducing it in equation (2.9) we can again derive relationships for *α* and 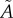 that only depend on the degradation rates (see the electronic supplementary material),2.10
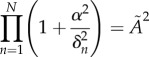
and2.11
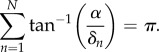


Note that in contrast with the three-gene case, there is no closed form expression for the angular velocity *α* and 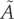 as a function of the degradation rates comparable to equations (2.4) and (2.5). Instead, we have the implicit equation (equation (2.11)) that returns the value of the angular velocity *α* for a certain set of values *δ*_*n*_ and equation (2.10) that returns the value of 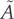, once *α* is known.

As in the previous section, we are interested in the degradation rates for which 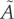 is minimized, since this will maximize the parameter region for which there will be oscillations. For this purpose, we can work with the arguments *θ*_*n*_ ≡ tan^−1^(*α*/*δ*_*n*_) varying independently in the domain [0, *π*/2) subject to the constraint that they sum to *π*. In this representation, 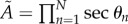 from equation (2.10). To find its minimum value, we minimize ln 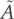 subject to the implicit equation (2.11) constraint (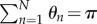) using the Lagrange multiplier *μ* (e.g. [34]),
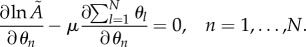
As 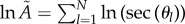, the minimization yields2.12
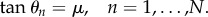
As *θ*_*n*_ vary in the domain [0, *π*/2), the condition (2.12) is only fulfilled when all *θ*_*n*_ are the same (*θ*_*n*_ = *π*/*N*). It is straightforward to check that this stationary point is a minimum since 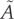 can be made arbitrarily big by choosing 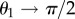. Thus, as in the classic three-gene repressilator, the minimum value of 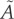 ≡ 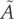
_*m*_ is achieved when all the degradation rates are equal. For this case, an analytical expression for 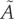 is available from equation (2.10),2.13
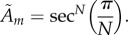
We can show that 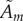 is decreasing in *N*, for *N* ≥ 3. Therefore, increasing *N* increases the range of values of *A* for which we get oscillations. As 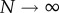, the critical value of *A* tends to 1, while when *N* = 3, the prediction 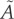
_*m*_ = 8 is recovered.

Additionally, an expression for the angular frequency *α* ≡ *α*_*m*_ of the small oscillations that arise close to this bifurcation point when all the degradation rates are identical *δ*_*n*_≡*δ* is also available,2.14
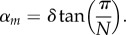
Like 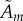, the frequency *α*_*m*_ is decreasing in *N* showing that the more links the feedback loop has, the slower the oscillations will get. For *N* = 3, the results from the first section are recovered, predicting an angular frequency is 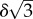, so that the time period of oscillations is 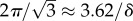. In the limit 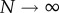, the transmission of information across the feedback gets infinitely slow and the frequency of the oscillations tends to 0. The slowing down of the oscillations with *N* is also true for the general case in which the degradation rates are not identical. If we fix *δ*_1_, *δ*_2_, …, *δ*_*N*_ and consider introducing an additional species *x*_*N*+1_ in the cycle (keeping the number of repressions odd), then it is clear from the implicit equation (equation (2.11)) that the value of *α* which satisfies this equation will be lowered.

**Figure 2.**
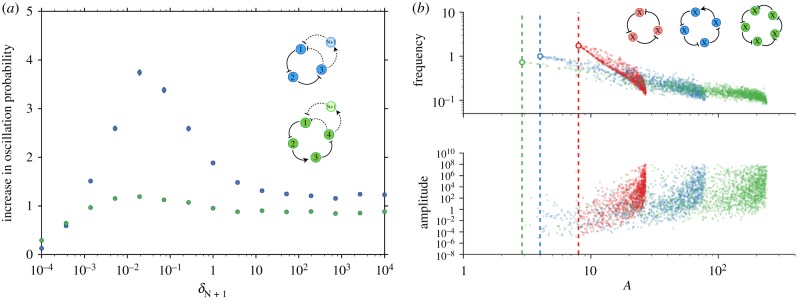
Behaviour of *N*-gene oscillators. (*a*) Ratio of the probability of oscillations in an extended ring network with *N* + 1 genes and a genetic network of *N* genes for different values of the degradation rate of the *N* + 1th gene. In both cases explored *N* = 3 (blue) and *N* = 5 (green), the degradation of the *N* + 1th gene is varied while sampling the other degradation rates from the range *δ*_*i*_ = [10^−3^, 1] and keeping one random gene fixed at *δ* = 1. The other parameters and details of the sampling are the same as in figure 1*c*. (*b*) Frequency and amplitude of oscillations as a function of the network parameter *A* for 1000 successfully oscillatory networks from a random screening for different numbers of species with the same degradation rates *δ*_*i*_ = 1, *N* = 3 (red), *N* = 4 (blue) and *N* = 5 (green). Dashed lines and rings show the minimum critical value 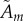 and angular velocity at that point *α*_*m*_ predicted by equations (2.13) and (2.14) for *N* = 3, 4, 5. The maximal values for *A* in the figure result from parameter sampling. In general *A*_max_ = (max ( Π*_i_ f_i_^'^*(x*))), which can be arbitrarily large. The repression functions are the same as in figure 1*c* with parameters logarithmically sampled from the intervals *k*_*i*_ : [10^−5^, 10^−1^], *a*_*i*_ : [10^−4^, 10^8^], *ρ*^*R*^_*i*_ : [10^−4^, 10^4^], *ρ*^*A*^_*i*_ : [10^−1^, 10^11^], *l*_*i*_ : [10^1^, 10^5^].

On the contrary, introducing a new species does not necessarily reduce 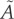. To evaluate the effect on 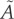 of adding a new link it is interesting to note first that in the case that the degradation rate of the new species tends to infinity (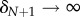 in equations (2.11) and (2.10)), the problem is reduced to the case with *N* species, i.e. the new variable will be so fast that it will always be in quasi-equilibrium with the previous species. By contrast, introducing an arbirtrarily slowly degrading species will completely stop the oscillations. It can be proved (see the electronic supplementary material) that there is a range of degradation rates of the new species for which the probability of oscillations is increased. This is supported by numerical simulations (figure 2*a*). The relative probability of oscillations does not precisely tend to one as the degradation rate of the added species tends to infinity, because the value of *A* is also changed by the addition of the extra species; in simulations in which the added species has *f*_*n*+1_(*x*) = *x*, the relative probability of oscillations tends to one as 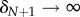 (data not shown). The simulations also demonstrate that the increase in probability of oscillations with additional species of intermediate degradation rate becomes weaker the more species there are.

### 2.3. Is the Hopf bifurcation supercritical or subcritical?

In the development of the argument, we assumed that the Hopf bifurcation is supercritical and not subcritical, i.e. a stable limit cycle arises at the bifurcation point. This is true for all the networks explored numerically in this manuscript, which use thermodynamic regulatory functions. Nevertheless, this is not necessarily true for any repressive functions *f*_*i*_(*x*). Mathematically, this requires the computation of the sign of the first Lyapunov coefficient [35] at the Hopf bifurcation. In the case where degradation rates and repressive functions are the same for all species (an assumption that is often made, e.g. [14]), progress can be made. For the three-gene repressilator with Hill function repressions it can be proved that the Lyapunov coefficient is negative, i.e. there is always a supercritical bifurcation [19]. In the case of an N-component repressive ring, the first Lyapunov coefficient ℓ_1_ is given by (see the electronic supplementary material),2.15
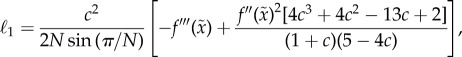
where *c* = cos(*π*/*N*). Therefore, the sign of ℓ_1_will depend on the ratio 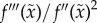 and the number of links. While the Lyapunov coefficient is negative for the thermodynamic regulatory functions chosen in this manuscript and for Hill function repressions, the Lyapunov coefficient is not negative for every possible regulatory function *f*. For example, a repressive ring with *f*(*x*) = 1/(1 + (1 + *x* − *x*^2^ + *x*^3^)^*h*^) can have a positive or negative coefficient depending on the exponent *h* (see electronic supplementary material, figure S1). Nevertheless, since trajectories for genetic systems are bounded, the unstable limit cycle must coexist with a stable limit cycle for *A* > *Ã*, returning a comparable set of results even in the case a subcritical bifurcation occurs. In this case, stable oscillations or evolution towards a steady state concentration will both be possible for values of *A* slightly lower than *Ã*.

As mentioned, the nature of the Hopf bifurcation depends on 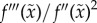. If it is greater than 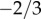, then the Hopf bifurcation is supercritical for all *N* for which it exists. If it is less than 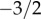, then the Hopf bifurcation is subcritical for all *N* for which it exists. If 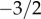, then the Hopf bifurcation is supercritical for sufficiently large *N* and subcritical for smaller *N*, assuming it exists.

### 2.4. Behaviour away from the Hopf bifurcation

We have shown that for values of *A* below the critical value (*A* < 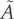), the steady state is stable, and changes stability at *A* = 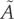. But, can the stability be recovered for greater values of *A*? Or in other words, is it guaranteed that the oscillations will be stable for all values *A* > 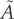
? To answer this question, we can count the maximum number of pairs of eigenvalues crossing the imaginary axis as the possible solutions of the implicit equation and also the number of pairs of eigenvalues with positive real part when 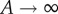 (see the electronic supplementary material). Strikingly, both magnitudes coincide, showing that every crossing of eigenvalues takes place from negative to positive real part, consequently the unstable state never recovers its stability and the oscillations are stable for every value of 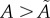.

Knowing that the system will be oscillating once *A* > 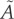 does not give information on the period or amplitude of the oscillations far from 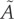. One possible approach to studying the frequency of the oscillations far from 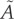 is to consider the imaginary part of the eigenvalues. If we consider the system with equal degradation rates, the characteristic equation (equation (2.8)) corresponds to2.16
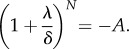
The eigenvalues are therefore given by 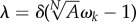 for *k* = 1, …, *N*, with *ω*_*k*_ the *N*th roots of −1. Thus, the eigenvalues with largest real part are 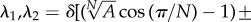 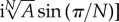. This confirms our result that the steady state is unstable for all *A* > sec^*n*^(*π*/*N*). In addition, perturbing around the steady state, the wavemode that grows fastest has frequency 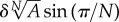. Although it is tempting to use this value as an approximation for the oscillation frequency far from the Hopf bifurcation, numerical simulations show that this fails to capture the full nonlinear behaviour. Instead they reveal a different scenario in which the oscillations can get slower as the value of *A* grows (figure 2*b*). On the other hand, as expected when moving away from a Hopf bifurcation, the oscillations gain amplitude as *A* increases (figure 2*b*).

### 2.5. Applications to mRNA and protein dynamics

So far we have been considered N-component repressive rings without taking any particular consideration of the nature of the biochemical species. It is interesting to focus on the case in which a negative repressive ring includes the mRNA and protein corresponding to each gene as different nodes of the regulatory network. In this scenario, the proteins regulate the mRNA production of other genes, while the mRNA of each gene is translated into the corresponding protein, keeping the same ring topology. Note that since protein translation always increases with the number of mRNA molecules, the number of repressions in the network is the same as for a network where the mRNA is not taken into account. Thus, the same theory developed in the manuscript applies with the difference that the number of nodes *N* is doubled and two temporal scales for the degradation of mRNA and protein are introduced, the latter being greater than the former.

One immediate observation is that a two gene negative feedback loop network without mRNA can never oscillate, since the minimum value of 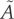
_*m*_ (equation (2.13)) tends to infinity for *N* = 2, making it impossible to find any set of parameters or regulatory functions able to make the system oscillate (*A* > 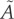
_*m*_). The same can also be seen from the implicit equation (equation (2.11)) where each of the two terms in the sum will always be less than *π*/2 for finite positive values of *α* and *δ*. By contrast, this is no longer true when mRNA is included in the description, since in this case, *N* = 4 and there is a finite value of 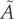
_*m*_ = 4, allowing the system to oscillate for values of *A* > 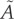 > 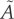
_*m*_, even though the gene network topology is the same. This explains the computational observations of Hazimanikatis & Lee [36], in which they study the danger of the common simplification of considering mRNA dynamics to be so fast that they can be considered in equilibrium. Concretely they observe that, for a two-gene feedback loop, considering mRNA to be at equilibrium extinguishes the oscillatory behaviour of the network. Not only can our analysis explain this behaviour, but it can also give a measure of the contribution of mRNA degradation to the oscillatory behaviour, indicating that the faster the degradation of the mRNA in comparison with the protein, the smaller will be the mRNA ‘angular’ contribution to the implicit equation (equation (2.11)).

**Figure 3.**
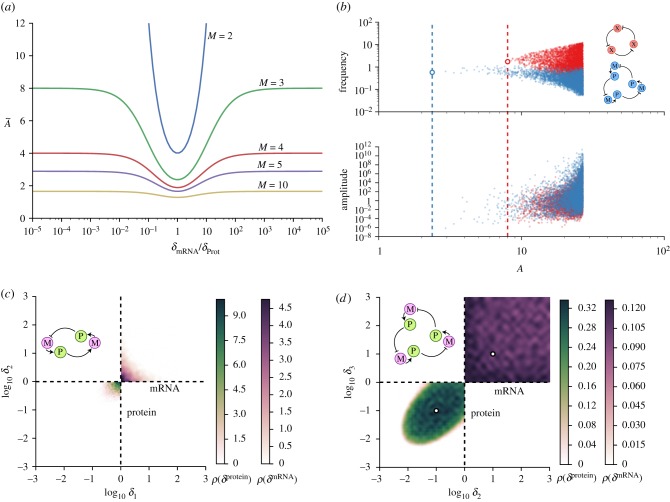
Effects of mRNA and protein degradation timescale differences. (*a*) Dependence of critical value of *A* on the ratio of the degradation rates of the protein and mRNA for a system composed of M genes with protein degradation rate *δ*_Prot_ and mRNA degradation rate *δ*_mRNA_. (*b*) Frequency and amplitude as a function of the network parameter *A* for 6000 successfully oscillatory networks from a random screening. The random screening was performed for the repressilator network simulated as direct repression between three genes (red) and as a six element network (blue) taking into account separately mRNA from protein dynamics. Dashed lines and rings show the minimum critical value 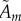 and angular velocity at that point *α*_*m*_ predicted by equations (2.13) and (2.14) for *N* = 3 and *N* = 6. Repression functions and parameter screening were the same as in figure 2*b* with additional screening on the degradation rates *δ*_Prot_ : [10^−3^, 1] and *δ*_mRNA_ : [1, 10^3^], keeping one of the degradation rates fixed as *δ*_Prot_1__ = 1. The translation of mRNA M into protein P is considered to be linear as *f*_*p*_(*m*) = *a*_*p*_*m*, where *a*_*p*_ was also logarithmically sampled (*a*_*p*_ : [1, 10^8^]). (*c*,*d*) Probability density of oscillations for the two (*c*)) and three (*d*)) gene network with mRNA (*M* = 2 and *M* = 3) for different sets of networks and degradation parameters. The degradation parameters of each test were sampled logarithmically from the ranges *δ*_mRNA_ = [1, 10^3^] (upper quadrant) and *δ*_Prot_ = [10^−3^, 1] (lower quadrant). Colours show the successfully oscillatory behaviour probability density of a network as a function of pairs of *δ*_mRNA_ and *δ*_Prot_. For the case *M* = 3, one of the species had fixed degradation rates given by *δ*_mRNA_1__ = 10 and *δ*_Prot_1__ = 0.1 (white circles). The random sampling of the other parameters of the network was the same as in figure 2*b*.

To understand what happens for a larger number of genes we consider the case where there are *M* genes composed by *M* mRNAs with degradation rate *δ*_mRNA_ and *M* proteins with degradation rate *δ*_Prot_, forming a negative feedback loop of *N* = 2*M* nodes in total. The critical value of *A* is given by equation (2.10),2.17
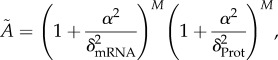
where the value of the angular velocity *α* is analytically available from the implicit equation (equation (2.11)), tan^−1^(*α*/*δ*_mRNA_) + tan^−1^(*α*/*δ*_Prot_) = *π*/*M* giving,2.18
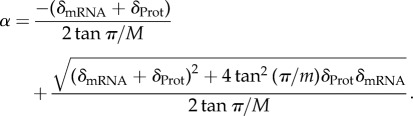
Substituting equation (2.18) into equation (2.17), it is straightforward to see that the value of 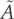 is solely determined by the ratio of degradation rates *δ*_mRNA_/*δ*_Prot_, and reaches a minimum of sec^*N*^(*π*/*N*), as expected, when the two degradation rates coincide (figure 3*a*).

In the specific case of the two node network of [36] (i.e. *M* = 2), we get2.19
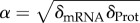
and the critical value of *A* is2.20
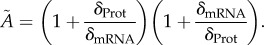
The nice simple form of equation (2.19) shows that at the bifurcation, oscillations occur on a timescale that depends on both mRNA and protein degradation rates and is intermediate between the two timescales. Additionally, as we have already discussed, equation (2.20) implies that 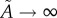 when 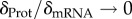. In particular, the steep variation of *Ã* with the ratio of the degradation rates makes it very difficult to find an oscillatory network when mRNA has a much faster degradation rate than protein, even when the condition is relaxed and the degradation rate of each of the four species is allowed to vary independently (figure 3*c*,*d*). We see in the figure that the four degradation rates need to be similar in order for oscillations to occur.

Interestingly, this strict condition does not apply to bigger networks. The dependence of 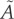 on *δ*_mRNA_/*δ*_Prot_ becomes less steep as *M* increases and 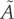 still takes a finite value of sec^*M*^(*π*/*M*) as 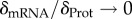 (figure 3).^1^ Similar things can be observed if we allow all the degradation rates to be different and we screen numerically for sets of degradation rates that give rise to oscillations (figure 3*c*,*d*). We see already for *M* = 3 that the actual values of the mRNA degradation rates are relatively unimportant (provided they are constrained to be higher than the protein degradation rates) and the possibility of oscillations is almost exclusively constrained by the degradation rates of the proteins, that are required to be similar.

As in the previous section, it is also interesting to study the behaviour of the oscillations far from the Hopf bifurcation when the mRNA is taken into account. As expected from the analysis, the introduction of new species slows down the system, yielding slower oscillations for all the values of *A* (figure 3*b*). Additionally, as was shown in the previous section, more species do not necessarily have an effect on the amplitude of the oscillations, which remain the same whether or not mRNA dynamics are considered.

## 3. Discussion

The results obtained in this study rely on working out properties of the eigenvalues of the system without determining exactly their values. Concretely, we find that the eigenvalues only depend on the values of the degradation rates and a single parameter *A* that summarizes all the topology, regulatory functions and specific parameters of the network. The power of this finding is that it allowed us to delve into details of the oscillatory behaviour that are universal for any repressive ring. The main conclusion deriving from this analysis is the requirement for identical degradation rates for all the genes in order to optimize the parameter space that allows oscillations. This property holds independently of how asymmetric the different regulatory functions are. Furthermore, it also yields a quantification of the range of heterogeneity among the degradation rates that can still allow oscillations. This information is valuable from the point of view of synthetic biology where fine tuning of the network is required to optimize oscillatory behaviour.

The limitations of these findings come in the indetermination of how different regulatory functions affect the actual values of the parameter *A*, suggesting a natural continuation of the research on the topic. Understanding how different biological parameters affect the value of *A* will lead to knowledge of how these parameters affect the properties of the oscillations of the system and how achievable is the oscillatory condition *A* > 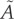. Similarly, we found that the approach fails to predict details of the oscillatory behaviour, such as frequency or amplitude far from the bifurcation point. Results show that for identical topologies, increasing *A* can lead to increasing or decreasing frequency, suggesting that further knowledge beyond *A* is required to address these questions.

One of the constraints of the current analysis is the requirement for directed interactions between genes, not allowing the direct inclusion of bidirectional interactions such as dimerization or promoter binding that can give rise to new bifurcations [25]. Nevertheless, in certain cases, careful analysis of the timescales can allow the equations to be rewritten preserving the sequentiality of the interactions and keeping similar symmetries to the ones studied in the current manuscript [37], indicating a possible extension of the current study to more precise repressilator descriptions.

Additionally, a recent study makes similar predictions regarding the homogeneity of the degradation rates in the more complex AC–DC network, consisting of a repressilator with an extra cross-repression where timescale separation is not possible [38]. Strikingly, for this network, optimization of the oscillatory behaviour revealed that, again, homogeneity of the degradation rates was required to observe oscillations. Such results hint at the possibility of extending our current analysis to more complex topologies.

Finally, the current study was limited to networks that involve a negative feedback loop. Nevertheless, there is also a body of research devoted to understanding the oscillations of positive feedback ring GRNs [18,21]. Even though the oscillatory orbits are unstable, they can show long-lived oscillations that allow fast controllable transients between oscillatory and non-oscillatory regimes [31]. The appeal of such networks also indicate a possible continuation of our work, seeking to understand the role of degradation rate homogeneity and number of nodes in the nature of such oscillations.

## Supplementary Material

Supplementary text

## Supplementary Material

Source code

## Endnote

1 In the limit as , both *Ã*_*m*_ and the value of *Ã* as  tend to one, so the dependence on *δ*_mRNA_/*δ*_Prot_ disappears.

## Data accessibility

The source code used for the simulations is provided as part of the electronic supplementary material.
