# Supplementary material for: Degradation rate uniformity determines success of oscillations in repressive feedback regulatory networks
Source: J R Soc Interface. 2018 May 9;15(142):20180157. doi: 10.1098/rsif.2018.0157 (PMC6000169; doi:10.1098/rsif.2018.0157)
Supplement: Supplementary text [file rsif20180157supp1.pdf]

Supplementary Material

Unattainability of chaotic regimes

One of the difficulties in extending the results to more than three dimensions is the possibility of chaotic attractors in the system. Nevertheless, monotone systems such as the ones described in equation (6), follow a Poincaré-Bendixson-type result [Mallet-Paret and Smith, 1990]. This implies that the ω-limit set of an orbit is either a steady state or a limit cycle. According to theorem 4.1 of [Mallet-Paret and Smith, 1990], the Poincaré-Bendixson-type result applies when $\mathbb{R}^n_+$ is positively invariant for monotone cyclic feedback systems defining a negative feedback loop that contain a unique critical point x^* with $\det(-J) > 0$. In our system, at the unique fixed point $\det(-J) = (1 + A)\prod_{n=1}^{N} \delta_n > 0$ (see eq. (9)). Additionally it is easy to show that $\mathbb{R}^n_+$ is positively invariant since at each hyperplane $x_n = 0$ the dynamics of the n-th component follows $\dot{x}_n = \delta_n f_n(x_{n-1}) > 0$ (see eq. (6)). As a result of this theorem, the system can only converge to a steady state or a limit cycle. The possible bifurcations in the N-dimensional space have a 2-dimensional analogue, excluding bifurcations such as period doubling. Therefore, the change of stability of any N-dimensional ring with $\prod_{n=1}^{N} \lambda_n \neq 0$ (see eq. (9)) can only correspond to a Hopf bifurcation.

Derivation of the Implicit Equation

Introducing the Hopf bifurcation condition $\lambda = i\alpha$ in Equation 9 we obtain,

$$\prod_{n=1}^{N}\left(1 + \frac{i\alpha}{\delta_n}\right) = -\widetilde{A}. \tag{S.1}$$

Taking the argument of both sides and using the identity $\sum_{i=1}^{n} \arg(z_i) \equiv \arg\left(\prod_{i=1}^{n} z_i\right)$, we obtain,

$$\sum_{n=1}^{N} \tan^{-1}(\alpha/\delta_n) = \pi + 2\pi k, \quad k = 0, 1, 2, ... \tag{S.2}$$

Similarly, taking the squared moduli of both sides of Equation S.1,

$$\prod_{n=1}^{N}\left(1 + \frac{\alpha^2}{\delta_n^2}\right) = \widetilde{A}^2. \tag{S.3}$$

The left hand side is increasing in α, so to find the smallest value of A for which there is a pair of imaginary eigenvalues, we must find the smallest α which satisfies Equation S.2. This will happen when the sum of the arguments equals π ($k = 0$), because each of the terms in the sum in Equation S.2 runs from 0 to $\pi/2$ as α increases. This reduces the Implicit Equation for α to

$$\sum_{n=1}^{N} \tan^{-1}(\alpha/\delta_n) = \pi. \tag{S.4}$$

Increase in oscillation probability after introducing a new species

The change of the parameter size for which the system oscillates as a function of the $N+1$th degradation rate of a ring of $N+1$ species can be obtained from Eq. 10 as

$$\frac{\mathrm{d}\ln\widetilde{A}}{\mathrm{d}\delta_{N+1}} = \frac{\alpha^2}{\delta_{N+1}(\delta_{N+1}^2 + \alpha^2)}\left[\frac{\sum_{n=1}^{N+1}\frac{\delta_{N+1}}{\delta_n^2+\alpha^2}}{\sum_{n=1}^{N+1}\frac{\delta_n}{\delta_n^2+\alpha^2}} - 1\right], \tag{S.5}$$

which means that $\frac{\mathrm{d}\widetilde{A}}{\mathrm{d}\delta_{N+1}} > 0$ if and only if $\sum_{n=1}^{N} \sin^2\theta_n(\cot\theta_{N+1} - \cot\theta_n) > 0$. Since increasing δ_{N+1}, while keeping the other degradation rates fixed, decreases θ_{N+1} and increases the other θ_n, every term in this sum is increasing in δ_{N+1}. This means that $\widetilde{A}$ decreases with δ_{N+1} until it reaches a certain value (which must be within the range of values of the other degradation rates). Above this value $\widetilde{A}$ increases. Thus the probability of oscillations increases from zero when δ_{N+1} is zero to a maximum value for some optimal value of δ_{N+1} (intermediate between the δ_n, $n = 1, \ldots, N$) and then decreases to the same probability as for the network without species $N+1$ as δ_{N+1} tends to infinity.

Persistence of instability for $A > \widetilde{A}$

The characteristic polynomial (Equation 8) is analytic in λ and A. Therefore by the implicit function theorem, the roots of the equation are analytic in A, except at the turning points of the polynomial. At these turning points it is straightforward to check that two negative real eigenvalues collide to form a pair of complex conjugate eigenvalues in such a way that the real and imaginary parts on each branch are continuous in A. The other eigenvalues are analytic in A at these points. We have already shown in the previous section that there are no zero eigenvalues for any value of A, therefore the real parts of an eigenvalue can only change sign as a pair of eigenvalues crossing the imaginary axis. From the multiplicity of the Implicit Equation (Equation S.2), we already saw that there will be other values of $\alpha_k > \alpha$ for which a pair of eigenvalues crosses the imaginary axis. In our analysis in the previous section we chose α to give the minimum value of $A = \widetilde{A}$. The other points where eigenvalues cross the imaginary axis will occur at $\widetilde{A}_k > \widetilde{A}$. This is clear from the LHS of Equations S.2 and 10, which are both increasing functions of α.

The RHS of the Implicit Equation (Equation S.2) has infinitely many possible values, i.e. $\sum_{n=1}^{N} \tan^{-1}(\alpha/\delta_n) = \pi, 3\pi, 5\pi, \ldots$. Nevertheless, each of the terms of the sum can contribute at most $\pi/2$ to the result. Therefore, the sum has the upper bound $\sum_{n=1}^{N} \tan^{-1}(\alpha/\delta_n) \leq N\pi/2$, and for finite α (necessary since A is finite), the inequality is strict. Therefore, when $N = 3, 4, 5, 6$ there can only

be one possible solution α of the Implicit Equation; when $N = 7, 8, 9, 10$ there can be two values of α, etc. Concretely, the maximum number of times there are eigenvalues crossing the imaginary axis is $\lfloor (N+1)/4 \rfloor$, where $\lfloor \cdot \rfloor$ is the *floor* operator.

Each time a crossing occurs, it must only consist of a single pair of eigenvalues. This can be seen by supposing the opposite case in which $i\alpha$ is a repeated root of the characteristic equation (Equation 8), $P(i\alpha) = \prod_{n=1}^{N}(1 + \frac{i\alpha}{\delta_n}) + A = 0$. In this case it will also be a zero of the derivative of the characteristic function $P'(i\alpha) = 0 = \sum_{n=1}^{N}(P(i\alpha) - A)/(\delta_n + i\alpha) = -A \sum_{n=1}^{N} \frac{\delta_n - i\alpha}{\delta_n{}^2 + \alpha^2}$, which is impossible since each term in the final expression has negative real part.

On the other hand, as $A \to \infty$, the characteristic equation (Equation 8) requires the moduli of the roots to be arbitrarily large, so that the value of λ/δ_n will eventually greatly outweigh 1 and λ will approach a value satisfying

$$\lambda^N = -A \prod_{n=1}^{n} \delta_n. \tag{S.6}$$

This has solutions $\lambda_k = \sqrt[N]{A \prod_{n=1}^{N} \delta_n} \omega_k$ with $k = 1, ..., N$, where $\omega_k = \mathrm{e}^{(2k-1)\pi i/N}$ are each of the N solutions to the Nth root of -1. Therefore, the number of eigenvalues with a positive real part will be the number of ω_k with $\arg(\omega_k) \in (-\pi/2, \pi/2)$, which for $N > 2$ is $2\lfloor (N+1)/4 \rfloor$. Thus, there are twice as many roots with positive real part as there are pairs of eigenvalues crossing the imaginary axis as A goes from 0 to ∞. This shows that every pair of eigenvalues crossing the imaginary axis must do so by changing the real part from negative to positive as A increases. Therefore, the steady state is unstable for all $A > \tilde{A}$.

Simulations of networks

The networks were simulated using Python custom code integrated using the PyDSTools dynamical systems environment [Clewley, 2012]. Trajectories were solved by precompiling in C a multistep Radau method. The source code has been made available as part of the Supplementary Material. Bifurcation diagrams were obtained using continuation techniques and integrations under the same Python environment.

First Lyapunov coefficient of the Hopf bifurcation

In the system with the same repressive function, f, for each species, the steady state $F(x^*) = x^*$ occurs when $f(x^*) = x^*$. A Hopf bifurcation occurs at $\tilde{x}$, where $f(\tilde{x}) = \tilde{x}$ and $f'(\tilde{x}) = -\sec(\pi/N)$.

In order to study the nature of the Hopf bifurcation, it is necessary to compute the First Lyapunov coefficient ℓ_1 evaluated at the critical point $\tilde{x}$, that can be obtained as [Kuznetsov, 2013],

$$\begin{aligned}\ell_1(\tilde{x}) \;&=\; \frac{1}{2\alpha}\Re\big[\langle p, C(q,q,\bar{q})\rangle - 2\langle p, B(q, J^{-1}B(q,\bar{q}))\rangle + \\ &\quad \langle p, B(\bar{q}, (2i\alpha I_N - J)^{-1}B(q,q))\rangle\big]\,,\end{aligned} \tag{S.7}$$

which involves the computation of inner products of vectors in $\mathbb{C}^N$: $\langle a, b\rangle = \bar{a}^T b$. The matrix J in Equation S.7 is the Jacobian of the dynamical system at the critical point. For the system with same repressive functions and degradation rates for each species this is given by

$$J = \begin{pmatrix} -1 & 0 & \dots & 0 & -\sec(\pi/N) \\ -\sec(\pi/N) & -1 & 0 & \dots & 0 \\ 0 & \sec(\pi/N) & -1 & 0 & \dots \\ \ddots & & & & \\ 0 & \dots & 0 & -\sec(\pi/N) & -1 \end{pmatrix}.$$

Note that for the sake of simplicity we have scaled the time with the degradation rate δ. Additionally, $\alpha = \tan(\pi/N)$ in Equation S.7 is the modulus of the pair of eigenvalues at the Hopf bifurcation and q the corresponding eigenvector

$$q = \begin{pmatrix} 1/\sqrt{N} \\ \omega/\sqrt{N} \\ \vdots \\ \omega^{N-1}/\sqrt{N} \end{pmatrix},$$

where $\omega = -\cos(\pi/N) + i\sin(\pi/N)$ is an Nth root of unity. On the other hand, p is the eigenvalue of J^T, corresponding to eigenvalue $-i\tan(\pi/N)$, that in this case is the same, $p = q$. The only missing ingredients to start computing $\ell_1(\tilde{x})$ are the definition of the bilinear and trilinear forms

$$B_j(y,z) = \sum_{k,l=1}^{N} \left.\frac{\partial^2 f_j}{\partial x_k x_l}\right|_{\tilde{x}} y_k z_l,$$

$$C_j(y,z,w) = \sum_{k,l=1}^{N} \left.\frac{\partial^3 f_j}{\partial x_k x_l x_m}\right|_{\tilde{x}} y_k z_l w_m \quad \forall j = 1, ..., N.$$

Now we can proceed by calculating sequentially the different terms of Equation S.7. The evaluation of the bilinear forms $B(q,q)$ and $B(q,\bar{q})$ is immediate,

$$B(q,q) = f''(\tilde{x})\begin{pmatrix} \omega^{2N-2}/N \\ 1/N \\ \omega^2/N \\ \vdots \\ \omega^{2(N-2)}/N \end{pmatrix}, \quad B(q,\bar{q}) = \frac{f''(\tilde{x})}{N}\begin{pmatrix} 1 \\ 1 \\ \vdots \\ 1 \end{pmatrix}.$$

Thus, the product $J^{-1}B(q,\bar{q})$ requires the knowledge of the row sums of J^{-1}. Since the row sums of J are the same and equal to $(-1 - sec(\pi/N))$, J is a stochastic matrix multiplied by $-1 - sec(\pi/N)$. Thus J^{-1} will also be a stochastic matrix multiplied by $(-1-\sec(\pi/N))^{-1}$, and its product with the bilinear form will be,

$$J^{-1}B(q,\bar{q}) = \frac{-f''(\tilde{x})}{N(1+\sec(\pi/N))}\begin{pmatrix} 1 \\ 1 \\ \vdots \\ 1 \end{pmatrix}.$$

So

$$B(q, J^{-1}B(q,\bar{q})) = f''(\tilde{x})\frac{-f''(\tilde{x})}{N(1+\sec(\pi/N))}\begin{pmatrix} \omega^{N-1}/\sqrt{N} \\ 1 \\ \omega\sqrt{N} \\ \vdots \\ \omega^{N-2}/\sqrt{N} \end{pmatrix},$$

and hence

$$\begin{aligned} \bar{p}^T B(q, J^{-1}B(q,\bar{q})) &= \frac{-f''(\tilde{x})^2}{N^2(1+\sec(\pi/N))}[N\omega^{N-1}] \\ &= \frac{f''(\tilde{x})^2}{N(1+\sec(\pi/N))}[\cos(\pi/N) + i\sin(\pi/N)]. \end{aligned} \tag{S.8}$$

Next, in order to compute the third term of Equation S.7 we need to work with the inverse $(2\tan(\pi/N)i - J)^{-1}$, which has rows which are permutations of each other as,

$$(2\tan(\pi/N)i - J)^{-1} = \begin{pmatrix} b_1 & b_2 & \ldots & b_N \\ b_N & b_1 & \ldots & b_{N-1} \\ b_{N-1} & b_1 & \ldots & b_{N-2} \\ \ddots & & & \\ b_2 & b_3 & \ldots & b_1 \end{pmatrix}.$$

So

$$\begin{aligned} &(2\tan(\pi/N)i - J)^{-1}B(q,q) = \\ &= \frac{f''(\tilde{x})}{N}\begin{pmatrix} b_1\omega^{2(N-1)} + b_2 + b_3\omega^2 + \ldots + b_N\omega^{2(N-2)} \\ b_N\omega^{2(N-1)} + b_1 + b_2\omega^2 + \ldots + b_{N-1}\omega^{2(N-2)} \\ \vdots \\ b_2\omega^{2(N-1)} + b_3 + \ldots + b_N\omega^{2(N-3)} + b_1\omega^{2(N-2)} \end{pmatrix} \\ &= \frac{f''(\tilde{x})}{N}[b_2\omega^{2(N-1)} + b_3 + \ldots + b_N\omega^{2(N-3)} + b_1\omega^{2(N-2)}]\begin{pmatrix} \omega^2 \\ \omega^4 \\ \ldots \\ \omega^{2(N-1)} \\ 1 \end{pmatrix} \\ &= [b_1 + b_2\omega^2 + \ldots + b_N\omega^{2(N-1)}]B(q,q). \end{aligned}$$

This means that $B(q,q)$ is an eigenvector of $(2\tan(\pi/N)i - J)^{-1}$ with eigenvalue $[b_1 + b_2\omega^2 + \ldots + b_N\omega^{2(N-1)}]$. Thus it must also be an eigenvector of $(2\tan(\pi/N)i - J)$ with eigenvalue $\frac{1}{b_1+b_2\omega^2+\ldots+b_N\omega^{2(N-1)}}$. On the other hand we know that,

$$(2\tan(\pi/N)i - J)B(q,q) = \frac{f''(\tilde{x})}{N}\begin{pmatrix} (2i\tan(\pi/N)+1)\omega^{2N-2} + \sec(\pi/N)\omega^{2(N-2)} \\ \vdots \end{pmatrix},$$

which means that the eigenvalue is

$$1 + 2i\tan(\pi/N) + \sec(\pi/N)\bar{\omega}^2,$$

therefore

$$B(\bar{q}, (2\tan(\pi/N)i - J)^{-1}B(q,q)) = f''\frac{f''(\tilde{x})}{N\sqrt{N}}[b_1 + b_2\omega^2 + \ldots + b_N\omega^{2(N-1)}]\begin{pmatrix} \bar{\omega}^3 \\ \bar{\omega}^2 \\ \bar{\omega} \\ 1 \\ \omega \\ \vdots \end{pmatrix}.$$

And the third inner product of Equation S.7 can be written as,

$$\begin{aligned} \bar{p}^T B(\bar{q}, (2\tan(\pi/N)i - J)^{-1}B(q,q)) &= \frac{f''(\tilde{x})^2}{N^2}[b_2\omega^{2(N-1)} + b_3 + \ldots + b_N\omega^{2(N-3)} + b_1\omega^{2(N-2)}]N\bar{\omega}^3 \\ &= \frac{f''(\tilde{x})^2\bar{\omega}^3}{N}\frac{1}{1+2i\tan(\pi/N)+\sec(\pi/N)\bar{\omega}^2}, \end{aligned}$$

which has a real part,

$$\Re\left(\bar{p}^T B(\bar{q},(2\tan(\pi/N)i - J)^{-1}B(q,q))\right) = \frac{f''(\tilde{x})^2}{N}\frac{c(2c-1)(2c^2-c-2)}{(1+c)(5-4c)},$$

where $c = \cos(\pi/N)$.
Finally, we need to use the trilinear form to compute the first term of Equation S.7,

$$C(q,q,\bar{q}) = \frac{f'''(\tilde{x})}{N\sqrt{N}}\begin{pmatrix}\omega^{N-1}\\ 1\\ \omega\\ \vdots\\ \omega^{N-2}\end{pmatrix}$$

and so

$$\bar{p}^T C(q,q,\bar{q}) = \frac{f'''(\tilde{x})}{N^2}N\bar{\omega} = \frac{f'''(\tilde{x})}{N}\bar{\omega}.$$

Putting the three terms together, the first Lyapunov coefficient is given by (with $c \equiv \cos(\pi/N)$):

$$\begin{aligned} l_1(\tilde{x}) &= \frac{1}{2\tan(\pi/N)}\Re\left[\frac{f'''(\tilde{x})}{N}\bar{\omega} + 2\frac{f''(\tilde{x})^2}{N(1+\sec(\pi/N))}\bar{\omega} + \frac{f''(\tilde{x})^2}{N}\frac{c(2c-1)(2c^2-c-2)}{(1+c)(5-4c)}\right] \\ &= \frac{1}{2N\tan(\pi/N)}\left[-cf'''(\tilde{x}) + \frac{-2c + \frac{(2c-1)(2c^2-c-2)}{(5-4c)}}{1+\sec(\pi/N)}f''(\tilde{x})^2\right] \\ &= \frac{c^2}{2N\sin(\pi/N)}\left[-f'''(\tilde{x}) + \frac{4c^3+4c^2-13c+2}{(1+c)(5-4c)}f''(\tilde{x})^2\right]. \end{aligned} \quad \text{(S.9)}$$

This quantity does not have a determined sign and therefore the Hopf bifurcation can be supercritical or subcritical depending on the details of $f(x)$. For instance, for the repressive function $f(x) = a/(1+(1+x-x^2+x^3)^h)$ the sign of ℓ_1 will depend on the coefficient h (see Fig.S.1).

The Hopf bifurcation is supercritical if and only if

$$-f'''(\tilde{x}) + \frac{4c^3+4c^2-13c+2}{(1+c)(5-4c)}f''(\tilde{x})^2 < 0. \quad \text{(S.10)}$$

The coefficient multiplying $f''(\tilde{x})^2$ is negative for all N and decreases monotonically from $-2/3$ for $N=3$ to $-3/2$ as $N \to \infty$. This means that the criterion on f at the fixed point necessary for supercriticality becomes less stringent as N increases, so the Hopf bifurcation (for a given f) is more likely to be supercritical as N increases.

If, as in [Buşe et al., 2009], we consider f to be a repressive Hill function, *i.e.* $f(x) = c/(1+x^r)$, then

$$\frac{-f'''(\tilde{x})}{f''(\tilde{x})^2} = \frac{r^2-15r+38}{2(r-5)^2}.$$

For $r > 2$, this is less than $2/3$ and therefore the Hopf bifurcation is supercritical for any $N \geq 3$. Note that we have shown that the Hopf bifurcation occurs when $f'(\tilde{x}) = -\sec(\pi/N)$, but for the Hill function system $f'(\tilde{x}) = -r\tilde{x}^r/(1+\tilde{x}^r)$, so $|f'(\tilde{x})| < r$, so for the Hopf bifurcation to occur requires $r > \sec(\pi/N) \geq 2$. Therefore the Hopf bifurcation only occurs for $r > \sec(\pi/N) \geq 2$ and is always supercritical. This extends the result of [Buşe et al., 2009] to the case of N species.

References

[Buşe et al., 2009] Buşe, O., Kuznetsov, A. and Pérez, R. A. (2009). Existence of limit cycles in the repressilator equations. International Journal of Bifurcation and Chaos *19*, 4097–4106.

[Clewley, 2012] Clewley, R. (2012). Hybrid Models and Biological Model Reduction with PyDSTool. PLoS Comput. Biol. *8*, e1002628.

[Kuznetsov, 2013] Kuznetsov, Y. A. (2013). Elements of applied bifurcation theory, vol. 112,. Springer Science & Business Media.

[Mallet-Paret and Smith, 1990] Mallet-Paret, J. and Smith, H. (1990). The Poincare Bendixson Theorem for Monotone Cyclic Feedback Systems. Journal of Dynamics and Differential Equations *2*, 367–421.

Supplementary Figures

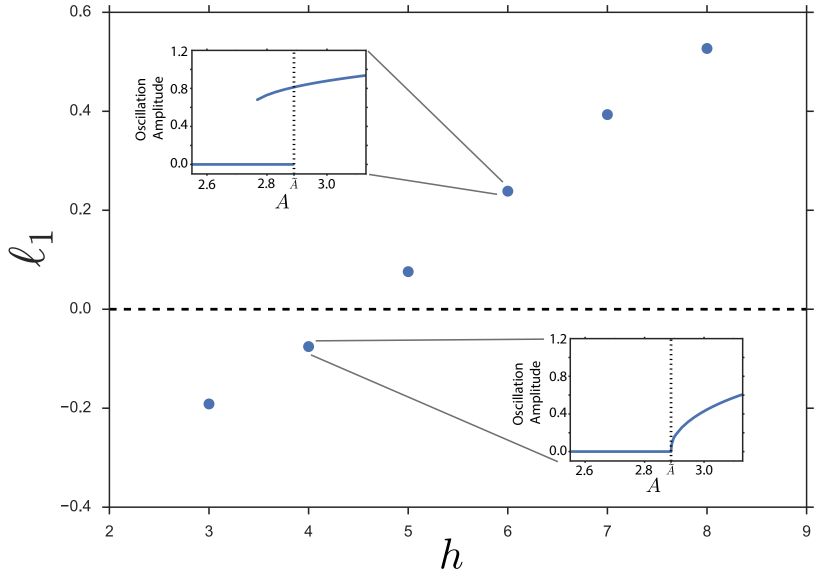

Figure S.1: *Depedence of sign of the first Lyapunov coefficient ℓ_1 with the exponent h for the repressive regulatory function $f(x) = a/(1+(1+x-x^2+x^3)^h)$ for a ring of 5 genes with identical degradation rates. Inset) Bifurcation diagrams for different values of ℓ_1 show how its sign determines if the Hopf bifurcation is supercritical or subcritical.*
